# Formulation and Characterization of Chitosan Films Incorporating Hawthorn Polyphenolic Extracts via Natural Deep Eutectic Solvents

**DOI:** 10.3390/polym17243250

**Published:** 2025-12-06

**Authors:** Oana Ciocirlan, Adina Gavrila, Gabriela Isopencu, Ludmila Motelica, Ovidiu-Cristian Oprea, Adrian Ionut Nicoara, Sergiu Sima, Paul Stanescu

**Affiliations:** 1 Faculty of Chemical Engineering and Biotechnologies, National University of Science and Technology Politehnica Bucharest, 132 Calea Grivitei, 010737 Bucharest, Romania; adina.gavrila@upb.ro (A.G.); gabriela.isopencu@upb.ro (G.I.); ovidiu.oprea@upb.ro (O.-C.O.); adrian.nicoara@upb.ro (A.I.N.); sergiu.sima@upb.ro (S.S.); paul.stanescu@upb.ro (P.S.); 2 Advanced Research Center for Innovative Materials, Products and Processes, National University of Science and Technology Politehnica Bucharest, 313 Splaiul Independentei, 060042 Bucharest, Romania; ludmila.motelica@upb.ro; 3 Academy of Romanian Scientists, 3 Ilfov Street, 050044 Bucharest, Romania; 4 Department of Science and Engineering of Oxide Materials and Nanomaterials, National Research Center for Micro and Nanomaterials, 313 Spl. Independenţei, 060042 Bucharest, Romania

**Keywords:** chitosan, biodegradable, vegetal extracts, polyphenols, hawthorn, density, viscosity

## Abstract

This study develops biodegradable chitosan (CS) films plasticized with natural deep eutectic solvents (NaDES) composed of choline chloride and glycolic acid (1:3 molar ratio). The same NaDES served as an effective extraction medium for bioactive compounds from hawthorn (*Crataegus monogyna*), which were incorporated into the chitosan matrix to enhance functionality. CS films with 44–70 wt% NaDES were evaluated, and the 50 wt% formulation exhibited the optimal mechanical and barrier performance. Upon extract incorporation, this film showed marked decreases in Young’s modulus (131→30 MPa) and tensile strength (24→12 MPa), relative to the extract-free counterparts, indicating enhanced flexibility. Stress–strain analyses confirmed a progressive reduction in stiffness with increasing NaDES content, evidencing its plasticizing effect. FTIR analysis revealed extensive hydrogen-bonding between CS and NaDES, alongside successful integration of polyphenolics extracted from hawthorn. Morphological analysis showed smooth, dense, homogeneous surfaces. Films exhibited strong UV absorption, with extract-loaded samples extending into the UVA and visible ranges, enhancing light-barrier properties. The presence of polyphenolic compounds enhanced the 1,1-diphenyl-2-picrylhydrazyl (DPPH) free radical scavenging activity to nearly twice that of the neat CS films. These combined mechanical, optical, and antioxidant properties highlight the potential of these NaDES-based chitosan films for sustainable active packaging.

## 1. Introduction

One of the most promising application areas for biodegradable natural polymers such as polysaccharides and their composites is the food packaging sector, where environmental sustainability can be achieved without major compromises in performance [1]. Among these materials, chitosan (CS) stands out as a particularly attractive biopolymer due to its combination of antibacterial activity, biodegradability, non-toxicity, and film-forming ability [2]. Moreover, CS can be obtained relatively easily from abundant natural sources and exhibits good thermal and chemical stability, making it highly suitable for the development of bio-based packaging films [3,4,5]. Compared to its parent compound, chitin, chitosan offers the additional advantage of solubility in mildly acidic aqueous media, which facilitates processing and formulation. Despite these benefits, the broader application of CS films remains limited by intrinsic mechanical drawbacks, including high rigidity, low flexibility, and relatively high water vapour permeability, resulting in modest barrier properties against gases and moisture. Consequently, significant research efforts are focused on identifying and integrating functional additives or plasticizers that can simultaneously enhance the mechanical strength, flexibility, and barrier performance of chitosan films, an ongoing challenge, as improvements in one property often compromise others [6,7,8,9,10].

Deep eutectic solvents (DES) are room-temperature liquid mixtures composed of a hydrogen bond acceptor (HBA) and a hydrogen bond donor (HBD), exhibiting remarkable physicochemical properties [11,12] and notable advantages such as low cost, simple preparation, and recyclability. In recent years, extensive research has focused on their application across diverse fields, primarily as environmentally friendly alternatives to conventional solvents [13,14,15]. The growing focus on sustainable technologies has further encouraged the utilization of renewable resources and the valorisation of waste materials through DES-based systems [16,17]. Moreover, natural deep eutectic solvents (NaDES), owing to their biodegradability, can play a dual role, serving both as efficient extractants of bioactive compounds from plant matrices and as plasticizers in polysaccharide-based films [18,19,20,21,22,23,24,25,26]. In active packaging, acidic NaDESs containing organic acids, such as the extensively studied lactic acid, serve as efficient plasticizers by forming hydrogen bonds between their acidic groups and the amino groups of chitosan, improving chain mobility and reducing film rigidity [27,28,29]. Overall, integrating NaDES with plant extracts into chitosan films led to significant enhancements in performance, particularly in antimicrobial and antioxidant activities [23,30,31]. Hawthorn is a bush with flowers, berries and leaves rich in polyphenols, flavonoids, and other active compounds [32] with many benefits for human health, which can be extracted in NaDES with very good results [33,34].

Therefore, the aim of this work is to exploit the dual role of NaDES as plasticizers and extraction solvents of polyphenols from hawthorn for the preparation of chitosan active films for food packaging. This study attempts to develop a novel NaDES system composed of choline chloride (ChCl) and glycolic acid (GA) in a 1:3 molar ratio, and benchmark its characteristics against ChCl-lactic acid (LA) system (1:1 molar ratio). The acidity provided by glycolic acid within the NaDES eliminates the need for prior dissolution of chitosan in acetic acid, as typically performed in standard procedures [29,35]. Chitosan films were prepared via the casting method by varying NaDES contents between 44 and 70 wt% and by adding hawthorn extract to tailor their mechanical, functional, and barrier properties. The influences of the extract, NaDES type and content on the thermal (TG, DSC) and structural (FTIR) properties of the chitosan films were evaluated. Mechanical performance was assessed through measurements of tensile strength (TS) and elongation at break (EB). The UV-blocking capacity of the films were investigated using UV–Vis absorption. Additionally, the films were characterized in terms of water vapour permeability (WVP). Given that antimicrobial and antioxidant properties are essential functional attributes of active packaging materials, specific assays were conducted to determine these activities. The antibacterial performance was tested against Gram-negative (*Escherichia coli*) and Gram-positive (*Bacillus subtilis*) strains, while the antioxidant capacity was evaluated via the DPPH radical scavenging method for both the films and the corresponding extracts. Additionally, new density and viscosity data for DES_GA3 in the 20–60 °C temperature range, reported here for the first time, were included.

## 2. Materials and Methods

### 2.1. Materials

Lactic acid (90% GPR RECTAPUR) and Glycolic acid (GA, purity ≥ 99 wt%, Carlo Erba, Cornaredo, Italy) were used without further purification. Choline chloride (ChCl, purity ≥ 98 wt%) and chitosan (CAS 9012-76-4, MW = 100–300 kDa, degree of deacetylation ≥75%, white powder) were obtained from Thermo Scientific Chemicals, Waltham, MA, USA, and used without further purification. 1,1-diphenyl-2-picrylhydrazyl (DPPH), gallic acid, ethanol (96%), and sodium carbonate were purchased from Sigma-Aldrich, St. Louis, MO, USA. Folin–Ciocalteu reagent was of analytical grade and was purchased from Merck, Darmstadt, Germany. Doubly distilled water was used in the preparation of NaDES extractant solutions and casting solutions. The hawthorn leaves and flowers used in this study originated from the same commercial batch supplied by Fares^®^ (Laboratoarele Fares Biovital SRL, Orăștie, Romania) and correspond to the plant material previously characterized in our earlier publication [32], ensuring botanical consistency and comparability between studies.

The antibacterial activity was evaluated against two reference bacterial strains: *Escherichia coli* (DH5K strain) and *Bacillus subtilis spizizenii Nakamura* (ATCC 6633). All microbial strains and materials were obtained from the Microorganism Collection of the Bioreactor Laboratory, Faculty of Chemical Engineering and Biotechnologies, National University of Science and Technology Politehnica Bucharest. Nutrient agar (Carl Roth, Karlsruhe, Germany) was used as the culture medium, as it is a standard medium suitable for the growth of non-fastidious microorganisms.

### 2.2. Preparation of NaDES

Choline chloride and glycolic acid/lactic acid were weighed on an analytical balance (HR-120 with a precision of ± 0.0001 g) and mixed in different molar ratios, ChCl:GA = 1:3 and ChCl:LA = 1:1, respectively, in a 50 mL vessel to obtain eutectic solvents. The mixtures were magnetically stirred for 1 h at 60 °C until homogeneous and colourless liquids were formed, which remained in a liquid state at 25 °C, as mentioned in the literature [36]. The notations of the obtained NaDESs are DES_LA1 (ChCl:LA = 1:1) and DES_GA3 (ChCl:GA = 1:3).

### 2.3. Characterization of NaDES

The studied NaDES was characterized for its physicochemical properties, including density and viscosity, over the temperature range of 20–60 °C. Density was measured using an Anton Paar DMA 4500 densimeter, Anton Paar GmbH, Graz, Austria (precision ± 0.00005 g·cm^−3^) via the oscillating-tube method. Viscosity measurements were carried out with an Anton Paar AMVn microviscometer, Anton Paar GmbH, Graz, Austria, based on the rolling-ball principle and equipped with a calibrated 4.0 mm capillary. The viscosity determinations exhibited reproducibility better than 0.5% and repeatability of ±0.1%. Both instruments featured integrated Peltier elements, enabling precise temperature control within ±0.01 K.

### 2.4. Preparation of CS–NaDES Films

**Figure 1:**
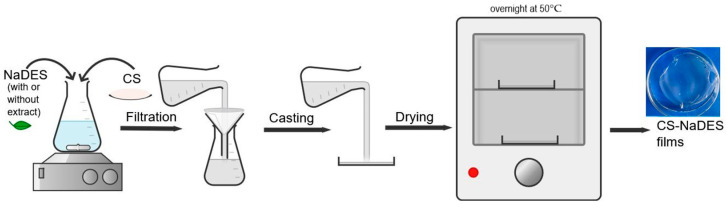
Schematic illustration of the preparation of CS-NaDES films.

CS films were fabricated via the casting technique (Figure 1) by dissolving chitosan in aqueous NaDES solutions containing ≤2% (*w*/*v*) total solids and ≤1% (*w*/*v*) chitosan, as detailed in Table S1. The newly developed CS films based on glycolic acid (DES_GA3) were prepared with varying NaDES contents of 44, 50, 60, and 70 wt%, whereas the comparative lactic acid-based film (DES_LA1) contained 50 wt% NaDES. The mixtures were magnetically stirred at room temperature for 8 h to achieve complete dissolution. The resulting casting solutions were clear and free of visible particulates or undissolved polymer; to ensure uniformity, all solutions were filtered before film casting. For the films containing extracts, NaDES was replaced with NaDES-extractant solutions, which served as the plasticizing agents.

The fresh filtered casting solutions were poured into Petri dishes, followed by drying in an oven at 50 °C overnight. After drying, the films were maintained in Petri and stored at room temperature (23 ± 2 °C) and relative humidity of 50 ± 3% for further investigations. The films were peeled off from the dishes before analysis. The notations of the obtained films are 44DES_GA3, 50DES_GA3, 60DES_GA3, 70DES_GA3, 50DES_GA3_ EH, 50DES_LA1, and 50DES_LA1_ EH.

### 2.5. Preparation of Extracts

For the extraction process, the microwave-assisted extraction (MAE) was used, which is a sustainable extraction method. Therefore, 30% (*w*/*w*) aqueous NaDES solutions were added to the plant powder at a plant-to-solvent ratio of 1:20 (*w*/*v*). The resulting mixtures were extracted using a Biotage Initiator reactor (Biotage AB, Uppsala, Sweden) equipped with magnetic stirring (900 rpm) and temperature control at 50 °C. The microwave power was maintained between 12 and 27 W, and the extraction time was 10 min. Following extraction, the mixtures were centrifuged (MC5000, LBX Instruments, Barcelona, Spain) at 4000 rpm for 10 min. The resulting supernatants were collected, transferred to clean plastic vials, and stored under refrigeration until analysis. We refer to these extracts as “NaDES-extractant solutions” and they are denoted as Ex_EH_GA3 and Ex_EH_LA1.

A detailed flowchart illustrating NaDES preparation, extract incorporation, and CS-film formation is provided in Figure S1.

### 2.6. Total Polyphenol Content

The total phenolic content (TPC) of the extracts was determined colorimetrically using the Folin–Ciocalteu method with minor modifications [32]. Fresh extracts were diluted 25-fold with distilled water, and 0.5 mL of the diluted solution was mixed with 5 mL of 10% (*v*/*v*) Folin–Ciocalteu reagent. After 5 min of stirring, 1.5 mL of 20% (*w*/*v*) sodium carbonate solution and 3 mL of distilled water were added. The mixtures were then incubated for 60 min in the dark at room temperature. Absorbance was recorded at 760 nm using a Shimadzu UV mini-1240 UV–Visible Scanning Spectrophotometer, Shimadzu Corporation, Kyoto, Japan (115 VAC). TPC values were expressed as milligrams of gallic acid equivalents per gram of dry plant material (mg GAE/g DM), based on a calibration curve constructed with gallic acid standards in the range of 1–5 mg/mL. The results were compared with those obtained using conventional solvents (water and 50% ethanol).

### 2.7. Antioxidant Properties

The antioxidant activity of the extracts and CS–NaDES films was assessed based on their DPPH radical scavenging capacity, following previously reported methods [37,38,39], with minor modifications.

For film samples, an ethanolic DPPH solution (0.071 mM) was prepared by dissolving 0.28 mg DPPH in 100 mL ethanol. Chitosan films (20 mg) were dispersed in 4 mL distilled water and homogenized by vortexing for several minutes; the resulting supernatant was collected. Subsequently, 1 mL of this solution was mixed with 4 mL of the DPPH solution and incubated for 30 min in the dark. The absorbance was then measured at 517 nm using a UV–Visible spectrophotometer.

The DPPH radical scavenging activity of the extracts was determined using the same protocol: 1 mL of extract was combined with 4 mL of DPPH solution, incubated in the dark for 30 min, centrifuged, and the absorbance of the supernatant was recorded at 517 nm. All measurements were performed in triplicate. The DPPH scavenging activity (%) was calculated using the following equation:

(1) $$\%DPPH=\;\frac{A^{DPPH}-A^{sample}}{A^{DPPH}}100$$

where $A^{DPPH}$ is the absorbance of the DPPH solution and $A^{sample}$ is the absorbance of the samples at 517 nm.

### 2.8. Antibacterial Activity

The following was determined: the antibacterial activity of the extracts and CS-NaDES films against Gram-negative bacteria *Escherichia coli* denoted EC, and Gram-positive bacteria *Bacillus subtilis spizizenii nakamura* denoted BS. The antibacterial activity of the film samples was evaluated in two experimental sessions, depending on the availability and freshness of the samples. Because the analyses were performed in two stages, the bacterial inoculum density was adjusted to 0.6 McFarland and verified using a UV–Vis spectrophotometer (uniSPEC 2). The corresponding optical density (OD) values were as follows: *E. coli*-0.2785 and *B. subtilis*-0.3015 for the tests involving extracts and neat films; *E. coli*-0.1827 and *B. subtilis*-0.1735 for the films containing extracts. Petri plates were inoculated with 100 µL of the bacterial suspension and incubated for approximately 1 h under controlled humidity conditions to allow uniform absorption of the inoculum into the medium and to avoid residual surface liquid.

For antibacterial testing, control film samples (CS films without extracts) were cut into square pieces of approximately 2 × 2 cm to evaluate the intrinsic antimicrobial effect of chitosan. Films containing plant extracts were cut into circular discs with a diameter of 6 mm, in accordance with the standard disc diffusion method [40]. All film samples were sterilized under UV light (256 nm) using a portable UV lamp (ROTH Type IV 254/366 nm) for 30 min and aseptically placed on the surface of the inoculated agar plates. The antibacterial activity of the extracts was assessed using the disc diffusion assay. Sterile paper discs were aseptically placed on the surface of the solidified, inoculated medium, and 6 µL of each extract was carefully pipetted onto the discs. All inoculated plates were incubated for 24 h at 37 °C, after which the inhibition zones were examined and measured.

### 2.9. Film Characterization

#### 2.9.1. FTIR Spectra

Fourier-transform infrared spectra for films, NaDES and their pure compounds were recorded with a Nicolet iS50 FTIR spectrometer (Thermo Fisher Scientific Inc., Waltham, MA, USA), with an ATR module, in the 4000–400 cm^−1^ domain, with a resolution of 2 cm^−1^; each spectrum was an average of 32 scans. FTIR 2D maps were recorded with a Nicolet iS10MX FTIR microscope (Thermo Fisher Scientific Inc., Waltham, MA, USA), in the 4000–650 cm^−1^ range.

#### 2.9.2. Thermal Properties

Thermal behaviour of films and pure NaDES was followed with a STA449C F3 system, TG-DSC (thermogravimetry–differential scanning calorimetry) from Netzsch (NETZSCH-Gerätebau GmbH, Selb, Germany), between 20 and 900 °C, in dynamic (50 mL/min) air atmosphere. The evolved gases were transferred through heated transfer lines and analyzed on the fly with the help of a FTIR Tensor 27 from Bruker (Bruker Co., Ettlingen, Germany), equipped with an internal thermostatic gas cell.

#### 2.9.3. UV-Vis and Fluorescence (PL) Spectra

A JASCO V560 spectrophotometer (JASCO Inc., Easton, PA, USA) was used to measure the UV–Vis spectra of CS films. The device was equipped with a 60 mm integrating sphere (ISV-469) and a film holder for the samples. The spectra were recorded with a speed of 200 nm min^−1^, in the domain of 200–900 nm.

A Perkin Elmer (Waltham, MA, USA) LS55 spectrometer was used to measure the photoluminescence spectrum (PL). A Xe lamp was used as a UV light source at ambient temperature, with the fluorescence being measured in the range of 350–800 nm. The spectra were recorded with a scan speed of 200 nm min^−1^, excitation and emission slits of 10 nm, and a 350 nm cut-off filter. An excitation wavelength of 320 nm was used.

#### 2.9.4. Scanning Electron Microscopy Analysis

The surface morphology of the samples was examined by scanning electron microscopy (SEM) using a Quanta Inspect F50 microscope (Thermo Fisher, Eindhoven, The Netherlands) equipped with a field emission gun (FEG) electron source, providing a resolution of 1.2 nm. The instrument was also fitted with an energy-dispersive X-ray spectroscopy (EDS) system offering a resolution of 133 eV at the MnK line. All images were acquired using the secondary electron detector (ETD) at an accelerating voltage of 30 kV, with a spot size of 3.5 and various working magnifications.

#### 2.9.5. Mechanical Test

The mechanical properties of the films were evaluated using an INSTRON universal testing machine, Darmstadt, Germany, equipped with rubber grips. Film samples were cut into rectangular strips of uniform dimensions (50 × 17 mm) and mounted between the grips with a gauge length of 20 mm. Tests were conducted at a constant crosshead speed of 5 mm/min, applying a pre-tension of 0.05 N. The instrument recorded the applied force (N) and corresponding deformation (%). Tensile strength (TS, MPa) and elongation at break (EB, %) were calculated from the stress–strain curves, while Young’s modulus (YM, MPa) was obtained from the initial linear region (0.05–2% strain) according to Hooke’s law. All measurements were performed in triplicate. Film thickness was determined at five random points using a precision micrometre (±0.001 mm).

#### 2.9.6. Moisture Content, Water Solubility and Water Vapour Permeability

The moisture content (MC) and water solubility (WS) of chitosan-NaDES films were determined according to the literature [21,41]. Film samples ($weight\;m_{0})$, cut into 20 × 20 mm pieces, were dried at 105 °C to constant weight ($m_{1}$) and subsequently immersed in 50 mL of distilled water for 14 h. The undissolved residues were then recovered and dried again at 105 °C to constant weight ($m_{2}$). Moisture content (MC) and water solubility (WS) were determined from the corresponding mass differences and were calculated as follows:

(2) $$MC\left(\%\right)=\frac{m_{0}-m_{1}}{m_{0}}\times 100$$

(3) $$WS\left(\%\right)=\frac{m_{1}-m_{2}}{m_{1}}\times 100$$

Water vapour permeability (WVP) of the chitosan films was evaluated using a gravimetric method. Films were mounted on vials (diameter 37 mm, height 90 mm) containing approximately 1.3 g of anhydrous CaCl_2_. The vials were initially weighed and then placed in a water desiccator maintained at 75% relative humidity and temperatures of 23 ± 2 °C. The mass of each vial was recorded at regular intervals over 2–3 days, ensuring that the CaCl_2_ remained in the solid state. The rate of water vapour transmission was determined from the slope of the mass change versus time plot, which was subsequently used in the WVP calculation according to the following equation:

(4) $$WVP=\;\frac{∆m\cdot d}{∆t\cdot A\cdot ∆P}$$

where ∆*m* is the weight gain (g), *d* is the film thickness (m), *A* is the area (m^2^) of the film surface, ∆*t* is the duration of exposure of the film to water vapour (s) and ∆*P* is the difference in partial pressure of water vapour on both sides of the film (Pa). All film analyses were performed in triplicate.

### 2.10. Statistical Analysis

The results obtained from mechanical tests, WVP, antioxidant activity and antibacterial activity analysis were expressed as mean values ± standard deviation (SD). Mean and SD were calculated with ANOVA (Tukey’s test at 95% confidence level), which was also used to establish whether there are significant differences between the films (*p* < 0.05).

## 3. Results and Discussion

The influence of NaDES content and extract on the molecular structure, morphology, thermal stability, optical and mechanical properties, photoluminescence (PL) spectra, and water vapour permeability of the films was studied. In addition, antimicrobial and antioxidant activities were evaluated, using CS films without extracts as a control. Additionally, the densities and viscosities of DES_GA3, as well as its thermal properties (TG and DSC) and Fourier transform infrared (FTIR) spectra, were determined.

We based our work on the well-established ChCl-LA (1:1) NaDES system [22,42,43]. We explored the effect of replacing lactic acid with glycolic acid, as this is a smaller molecule, expected to generate a denser and more cohesive CS–NaDES network, thereby enhancing the water-barrier properties. The better candidate, with respect to mechanical properties, was the 1:3 formulation, which was also of interest from an extraction standpoint due to its higher acidity.

### 3.1. FTIR Spectra Results

FTIR spectroscopy was used to gain information about the molecular structure of CS–NaDES films. Also, the structures of pure NaDES, extract, and chitosan were analyzed.

**Figure 2:**
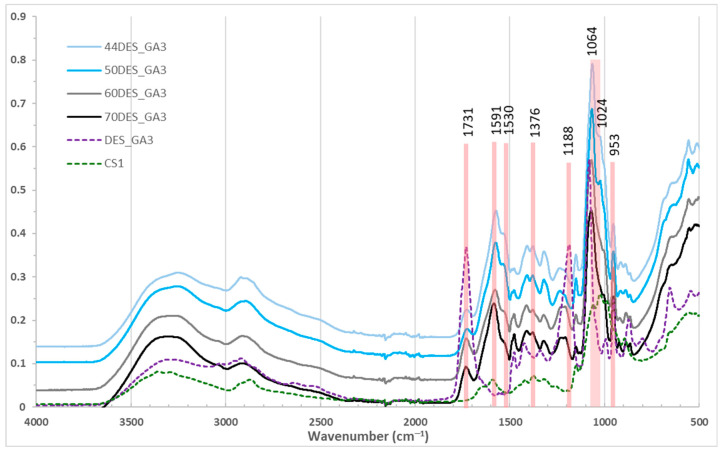
FTIR spectra for chitosan films in DES_GA3 with different NaDES content.

**Figure 3:**
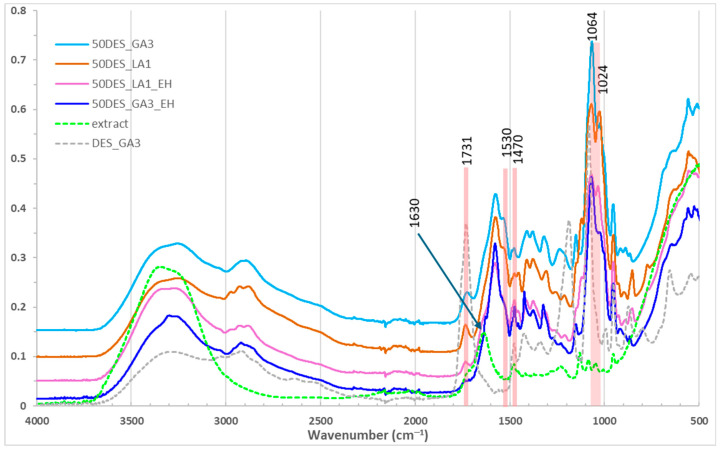
FTIR spectra for films with 50% NaDES, with and without hawthorn extract.

FTIR spectra of chitosan films [44,45] with varying NaDES content are shown in Figure 2 and Figure 3. Broad bands around 3250 cm^−1^ correspond to overlapping –OH and –NH stretching vibrations from chitosan and NaDES [23]. The N–H bending band of chitosan at 1591 cm^−1^ shifted to 1571–1580 cm^−1^ in films, with intensity increasing as NaDES content decreased, indicating protonation of chitosan by the organic acids. Similarly, the –CH_3_ deformation band at 1376 cm^−1^ shifted to 1370 cm^−1^. The amide I band at 1653 cm^−1^ [46] is no longer observed in the films and is replaced by a peak around 1530 cm^−1^, attributed to –NH_3_^+^ vibrations, whose intensity increases as the NaDES content decreases [22]. C–O–H and C–O–C vibrations at 1064 and 1024 cm^−1^ were intensified, suggesting new intermolecular interactions and inclusion of glycolic/lactic acid in the chitosan matrix.

The C=O stretching peak of glycolic/lactic acid at 1730 cm^−1^ decreased with NaDES content (Figure 2), reflecting conversion to carboxylate, while C–C–O vibrations at 953 cm^−1^ slightly diminished. Films with 50% NaDES (DES_GA3 vs. DES_LA1) showed similar peak positions (Figure 3) [22], but slightly higher intensity for DES_GA3, consistent with its higher acidity and COOH content. Also, a shift in the peak at 1188 cm^−1^ (C–O) stretching of NaDES toward higher wavenumbers is observed, accompanied by a decrease in intensity when its content increases in the CS films.

Comparing pure NaDES with NaDES extract solutions revealed similar spectra, except for an enhanced O–H stretching band at 3300 cm^−1^ in extracts due to polyphenols. Also, other characteristic peaks of polyphenols are observed in the NaDES extractant solution, at 1630 cm^−1^ (C=C stretching of aromatic ring), and at 1470 cm^−1^ (–CH_3_ bending), confirming aromatic and polyphenolic incorporation [21,47,48]. The characteristic COOH peak (of GA) at 1730 cm^−1^ is smaller in extract, a sign that new hydrogen bonds are established between GA/LA with the polyphenols in the extract.

In films with NaDES extracts (Figure 3), similar patterns were observed, with subtle changes such as disappearance of N–H and O–H bending shoulder from 1530 cm^−1^ and decreasing the band from 1024 to 1064 cm^−1^. Also, the characteristic peak of polyphenols at 1630 cm^−1^ diminished to a shoulder in DES_GA3_EH and disappeared in DES_LA1_EH film. These results demonstrate that NaDES effectively mediate polyphenol incorporation into chitosan films while modulating intermolecular interactions.

Moreover, the homogeneity of the films was monitored by FTIR microscopy. Therefore, FTIR maps for the chitosan films with different NaDES content are shown in Figure S2. It can be observed that the maps recorded at specific wavelengths for chitosan and NaDES indicate a good homogeneity at micron level. Also, the map for the film with hawthorn extract indicates that the plant extract in NaDES was successfully dispersed and incorporated in the chitosan film.

### 3.2. Thermal Properties Results

**Figure 4:**
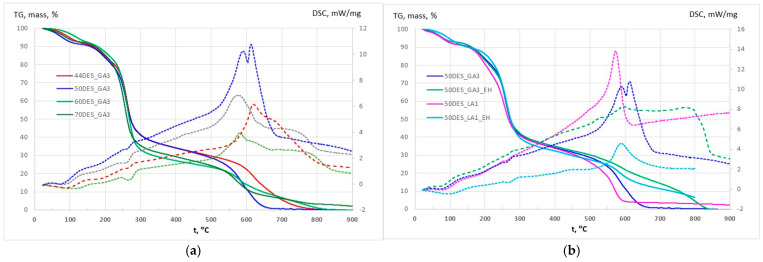
TG (solid) and DSC (dashed) curves for (**a**) CS films with different NaDES content in DES_GA3 and (**b**) CS films with hawthorn extracts in DES_GA3 and DES_LA1.

CS–NaDES films, with and without plant extracts, were characterized by TG and DSC (Table S2; Figure 4a,b). All films exhibited similar thermal behaviour, with stability up to 140–170 °C and maximum decomposition between 253 and 265 °C. An initial 9–11% weight loss between 70 and 170 °C corresponds to the removal of free and bound water, accompanied by a weak endothermic signal with a minimum between 70.2 and 110.3 °C (Endo I peak in DSC curves). The main decomposition (170–400 °C) involves overlapping reactions, including polymer–NaDES network fragmentation and partial oxidation, showing endothermic peaks (Endo II peak in DSC curves) that intensify with higher NaDES content, consistent with increased mass loss. This led to the sinusoidal aspect of DSC curve in the 170–400 °C interval. The general trend is exothermic, but it has a clear endothermic peak (Endo II), indicating the dominance of oxidation and fragmentation reactions at different temperatures. Above 400 °C, slow mass loss continues up to 550–650 °C, culminating in strong exothermic oxidation of residual carbonaceous material, with peak temperatures decreasing from 620 to 578 °C as NaDES content rises (Exo peak in DSC curves in Figure 4a). The mass loss observed in this temperature interval increases as the NaDES content decreases (from approximately 27% to 35%). The addition of choline chloride (higher NaDES percent) lowers the onset of chitosan decomposition (270–290 °C for pure CS), indicating a plasticizing effect (Figure 4a).

Overall, thermal behaviour is influenced by NaDES composition, with higher NaDES promoting earlier degradation of the polymer backbone but enhancing flexibility through plasticization. Films containing extracts (Figure 4b) exhibited the same three-stage degradation pattern as films without extracts. Upon incorporation of the extract, water evaporation shifts to higher temperatures compared with films containing the same NaDES level. This behaviour can be attributed to the presence of hydrophobic compounds in the extracts, which enhance the thermal stability of the films.

The thermal behaviour was similar across samples, with the exception of DES_LA1 films, which appeared slightly less stable. The incorporation of the EH into the films generates additional carbonaceous mass, originating from polyphenols and other aromatics, which require higher temperature for complete oxidation and removal.

### 3.3. UV-Vis and Fluorescence (PL) Spectra Results

**Figure 5:**
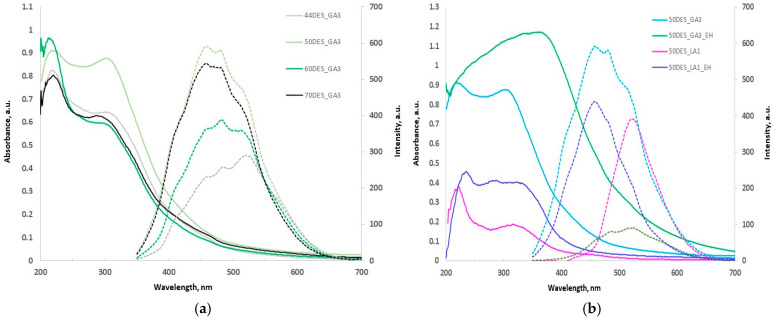
UV-Vis absorption (solid) and PL (dashed) spectra for (**a**) CS films with different NaDES content in DES_GA3 and (**b**) CS films with hawthorn extracts in DES_GA3 and DES_LA1.

Chitosan films plasticized with glycolic acid-based NaDES exhibit strong UV absorption and notable visible emission (Figure 5a,b). Films containing DES_GA3 show intense absorption in the UV region (220–350 nm), with the 220 nm peak dominating with increasing NaDES content, while the 310 nm band diminishes to a shoulder. The 50DES_GA3 film displays the largest UV band, whereas in 70DES_GA3, this band decreases.

Incorporation of plant extracts (Figure 5b) broadens the absorption band from UVA to UVB and shifts the maximum to ~390 nm, particularly for DES_GA3 films, likely due to polyphenolic compounds. DES_LA1 films show modest absorbance in the UV region. Overall, CS-DES_GA3 films loaded with hawthorn extracts demonstrate excellent UV barrier properties, highlighting their potential for food packaging applications.

The chitosan-based films exhibit broad visible photoluminescence, shifting from blue to green as the NaDES content decreases from 70% to 44%. Films with higher NaDES concentrations display stronger emission. Three distinct peaks are observed at approximately 455, 480, and 525 nm. Incorporation of plant extracts leads to quenching of fluorescence, likely due to electron-rich polyphenols providing alternative non-radiative de-excitation pathways.

### 3.4. Scanning Electron Microscopy Analysis Results

**Figure 6:**
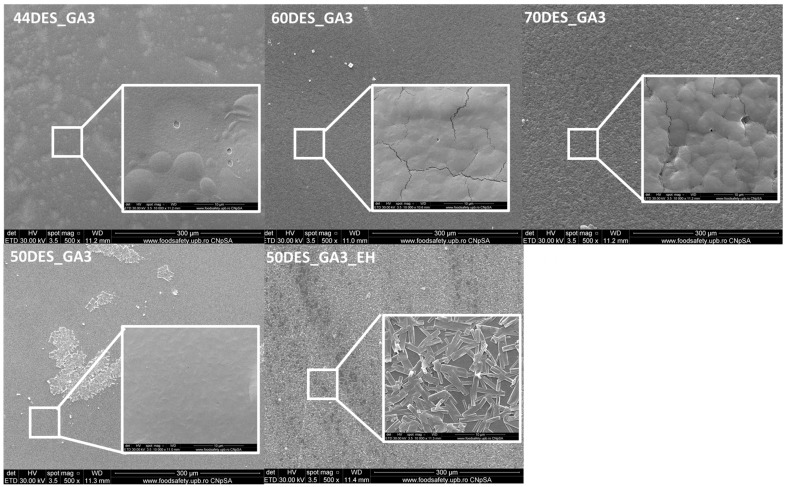
Surface images of CS films using scanning electron microscopy (SEM) at magnifications of 500× and 10,000×.

Microstructural characterisations obtained by scanning electron microscopy (Figure 6) reveal similar surface characteristics of the CS–NaDES films. The surface is continuous, uniform, without cracks or bubbles, but with small, isolated pores, with a diameter below 250 nm. Similar findings are reported in the literature [49]. However, when the NaDES content exceeds 60%, slight structural modifications occur in the polymer structure, promoting their organization into spherical particles with diameters of 2–4 µm (as observed for 70DES_GA3) [30]. In the case of samples containing hawthorn extract (50DES_GA3_EH), interconnected polyhedral crystals with widths ranging from 0.5 to 2.2 µm were observed on the surface of the chitosan film.

### 3.5. Visual Appearance and Mechanical Properties Results

The films obtained are homogenous, without cracks or bubbles, from transparent to slightly opalescent when the content of NaDES increases (Figure S3). Adding extracts gave the films a light brown colour. Because the materials allow the food product to be viewed, they can be accepted by the consumer, which is a plus.

**Figure 7:**
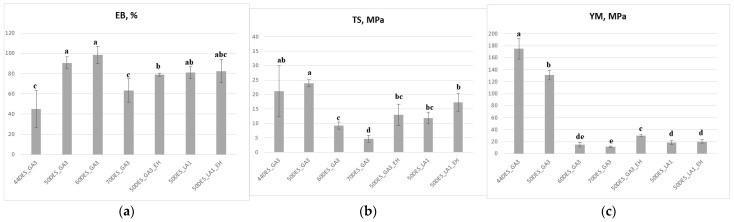
Influence of NaDES type and content, and extract addition, on (**a**) EB; (**b**) TS; (**c**) YM; different letters indicate statistically significant differences between films (*p* < 0.05).

As shown in Figure 7a, increasing the NaDES content enhanced the films’ flexibility and elasticity, as reflected by an increase in elongation at break (EB) up to 60% NaDES, beyond which EB decreased, consistent with observations by Sokolova et al. [24]. EB values ranged from 45% for 44DES_GA3 to approximately 100% for 60DES_GA3. The incorporation of EH extract caused a slight reduction in EB (*p* < 0.05) compared to the corresponding 50% DES films. Overall, the EB value of 80% recorded for 50DES_GA3_EH is sufficiently high to ensure good film flexibility. DES_LA1-based films, with or without extract, exhibited similar EB values, around 80%, with no significant differences between them (*p* > 0.05). Films containing 60–70% NaDES were softer and more fragile, with tensile strength (TS) around 5–8 MPa, whereas films with higher chitosan content were more rigid, showing TS greater than 20 MPa (Figure 7b).

Analysis of tensile curves (Figure S4) showed that YM, which reflects film rigidity, decreased with increasing NaDES content (Figure 7c), confirming the plasticizing effect of NaDES. The highest YM was recorded for 44DES_GA3 (~176 MPa), while DES_LA1 films had YM around 20 MPa. Overall, increasing the NaDES fraction to 70 wt% decreased YM to approximately 12 MPa, while the addition of EH extract consistently produced significantly lower YM values than extract-free films (*p* < 0.05), representing a ~80% reduction. It was observed that in 50DES_GA3_EH film, a reduction in Young’s modulus from 131 to 30 MPa and a reduction in tensile strength from 24 to 13 MPa occurs. In contrast, DES_LA1-based films exhibited the opposite behaviour, with tensile strength increasing upon EH incorporation. These results support their applicability in food packaging, such as for fresh-cut dried raw salami.

### 3.6. Moisture Content, Water Solubility and Water Vapour Permeability Results

The moisture content (MC) and water solubility (WS) showed an increasing trend with increasing NaDES content (Table 1). For instance, MC ranged from 17.5% in 44DES_GA3 to 24.7% in 70DES_GA3 films. Also, WS increases correspondingly from ~32% for 44DES_GA3 to 50.5% for 70DES_GA3 film. This can be explained by the increased ability of the hydrogen bond network in the chitosan matrix with high DES content to retain water [50]. EH extract led to a slight increase both in MC and WS compared with the corresponding 50% NaDES formulation.

**Figure 8:**
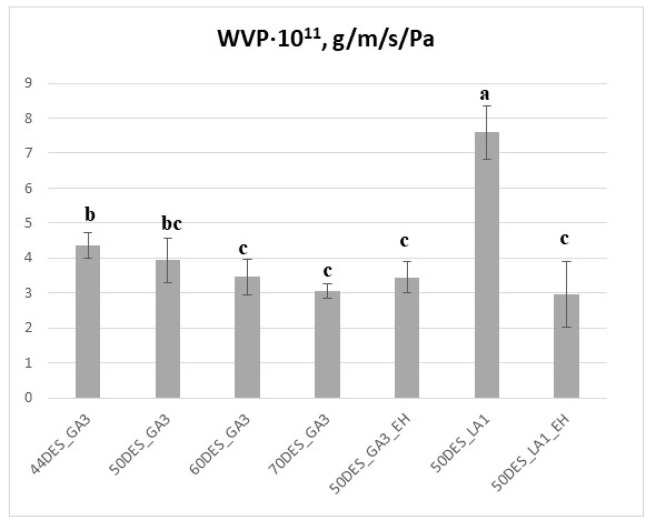
Water vapour permeability of films as a function of NaDES content and extract addition; different letters indicate statistically significant differences between films (*p* < 0.05).

Chitosan–NaDES films generally exhibit increased water vapour permeability (WVP) due to the hydrophilic nature of DES [23,43]. Incorporation of hydrophobic components, either via hydrophobic DES or extracts, reduces WVP, a desirable property for food packaging [38,50,51]. Although films with higher DES content retain more water, this does not necessarily correspond to increased water vapour permeability, as indicated by the data shown in Figure 8. WVP values for DES_GA3-based films ranged from 3 to 4.6 × 10^−11^ g·m^−1^s^−1^Pa^−1^, whereas DES_LA1 films exhibited higher WVP, exceeding 7 × 10^−11^ g·m^−1^s^−1^Pa^−1^. These effects arise from hydrogen-bonding interactions between chitosan chains and NaDES components, which, together with the smaller glycolic acid molecules, promote the formation of a denser and more cohesive CS–NaDES network, thereby lowering the water vapour permeability of the resulting films. This structural compactness also correlates with the higher thermal stability observed for DES_GA3 films compared to DES_LA1, indicating consistency between WVP and thermal analysis results.

Addition of EH extract slightly but significantly reduced WVP for DES_GA3 films (*p* < 0.05). A more pronounced reduction was observed for DES_LA1 films upon extract addition, with favourable WVP values (~3 × 10^−11^ g·m^−1^s^−1^Pa^−1^), comparable with literature reports [22,38]. This decrease is likely due to the presence of hydrophobic compounds filling interstitial spaces in the film matrix, thus reducing water vapour transmission. Overall, increasing NaDES content (from 44 to 70%) resulted in a modest further reduction in WVP, confirming the combined effects of compact structure and hydrophobic interactions in controlling water vapour transmission. Thus, the relatively small WVP values for DES_GA3 films offer a favourable premise for the development of chitosan films intended for dry-food packaging.

### 3.7. Total Polyphenol Content, Antioxidant and Antibacterial Properties Results

**Figure 9:**
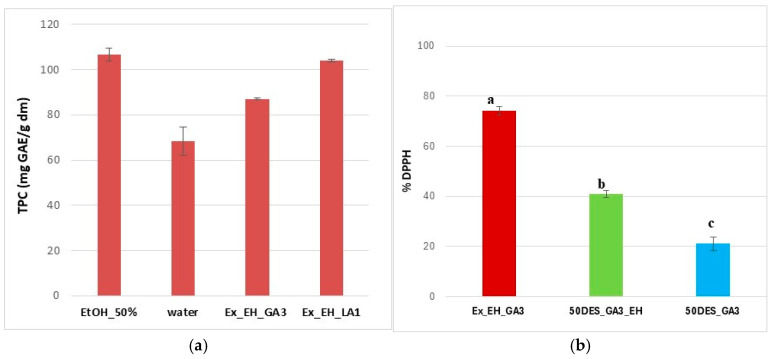
TPC content (**a**) and DPPH free radical scavenging activity (**b**) for extracts (brown), films with (green) and without (blue) extracts in different NaDESs; different letters indicate statistically significant differences between films (*p* < 0.05).

Our preliminary investigations (not shown here) aimed to identify the NaDES compositions that yield the highest total phenolic content (TPC) compared to water and 50% ethanol references. The results indicated that TPC values exceeding 100 mg GAE/g DM were achieved using 30–50% aqueous DES_LA1 systems. Consequently, 30% aqueous NaDES solutions were selected as medium for hawthorn extraction in DES_GA3, offering a balanced compromise between a slightly lower TPC value (87 mg GAE/g DM; Figure 9a) and the notable safety and environmental advantages of NaDES over toxic ethanol. When compared with literature data obtained under optimized conditions using similar NaDES formulations, the TPC values achieved in this study are highly competitive and demonstrate promising extraction performance [52].

The TPC obtained in the present study aligns well with our previously published results on hawthorn extracts [32], where detailed HPLC profiling under optimized extraction conditions yielded TPC values ranging from 93 to 110 mg GAE/g DM. Thus, the quantified levels of key phenolics—such as gallic acid, chlorogenic acid, vitexin, hyperoside, and quercetin—confirmed that hawthorn typically exhibits a phenolic richness consistent with the overall extraction yields observed here. The proximity of the TPC values between the two studies supports the reliability of the current extraction approach and demonstrates that NaDES-based extraction achieves phenolic recoveries comparable to conventional ethanol-based methods, while offering a greener and safer solvent system.

Antioxidant activity in extracts is indicated in Figure 9b, where EH extracts have a DPPH inhibition of 74% in DES_GA3 solution. As can be seen, the addition of extracts caused an increase in the antioxidant activity of the films of over 40%, compared to films with the same level of NaDES, as is observed in the literature [53]. Therefore, these results are in concordance with those obtained from UV-Vis absorption spectra. It can also be seen that the incorporation of extracts into the films resulted in a reduction in the percentage of DPPH inhibition compared to the extracts, as expected.

The results of antimicrobial activity are expressed as the zone of inhibition (IZ, mm) by the clear zone that differentiates around the disc or film with the active substance (Table 2). The precipitation that occurs when chitosan is added to NaDES solutions with extracts is probably due to a colloidal association between the positively charged chitosan and the negatively charged components/proteins present in the extract. This aspect influences sampling and the results of antimicrobial activity.

Analysis of the results indicates that the DES_GA3 matrix, when used as a support for hawthorn extract incorporation, produced a larger inhibition zone compared to the corresponding DES_LA1 system. As shown in Table 2, the extract prepared in DES_GA3 (Ex_EH_GA3) exhibited higher antimicrobial efficiency than Ex_EH_LA1 against both tested bacterial strains.

Among the cast films with extracts, the 50DES_GA3_EH sample demonstrated antibacterial activity against the Gram-negative *E. coli* strain. In contrast, Gram-positive *B. subtilis* showed lower susceptibility, not detected in films with extracts. This reduced sensitivity can be attributed to the structural characteristics of Gram-positive bacteria, namely their thicker peptidoglycan cell wall, which limits the diffusion of antimicrobial compounds. Consequently, a higher concentration or improved release of active substances would be required to achieve comparable inhibition.

Among the cast films without extracts, the 50DES_LA1 film demonstrated a higher antibacterial activity against the Gram-positive *B. subtilis* strain, more important than for the 50DES_GA3 sample. These findings are consistent with the DPPH radical scavenging results (Figure 9), confirming the correlation between antioxidant and antimicrobial activities [54].

### 3.8. Characterization of DES_GA3 

The densities and viscosities of pure NaDES DES_GA3, reported here for the first time in the literature, along with its thermal behaviour (TG and DSC) and Fourier transform infrared (FTIR) spectra, were analyzed.

**Figure 10:**
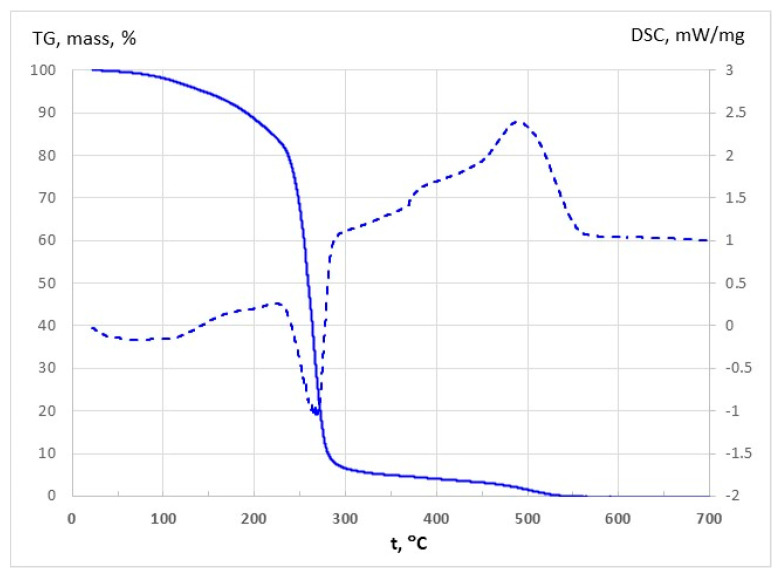
TG (solid) and DSC (dashed) curves for pure DES_GA3.

The thermal stability of pure DES_GA3 was investigated by TG and DSC curves and the results are shown in Table 3 and Figure 10. Analysis of the TG curves confirms a water loss of approximately 2% over the RT-100 °C range, which confirm the water content of 2 wt% in liquid state. The mass loss is about 16% over RT-220 °C interval and corresponds to the decomposition temperature of 227.4 °C. Further, by increasing temperature, most significant mass loss occurs (around 78%) which corresponds to endothermic process around 268 °C. The second exothermic process (Exo II) leads to a small mass loss and occurs at 489 °C.

Therefore, for the purposes of our study, the temperature range RT-220 °C ensures very good thermal stability for pure DES_GA3 which is attributed to the intermolecular interactions between ChCl and GA. Jablonský et al. also note long-term stability for ChCl-GA deep eutectic solvents, which recommend the potential practical use of this NaDES in many industrial applications [55].

**Figure 11:**
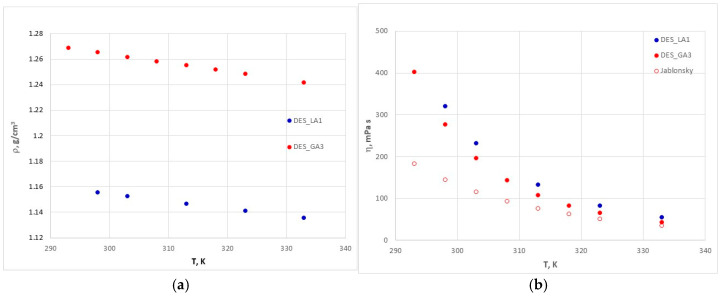
Density (**a**) and viscosity (**b**) of pure DES_GA3.

The densities and viscosities of pure DES_GA3 over the temperature range 20–60 °C, shown in Figure 11, are reported for the first time in the literature, to the best of our knowledge. The density decreases linearly with temperature and viscosity decreasing exponentially. It can be observed that DES_GA3 is slightly less viscous, and its density is higher than for DES_LA1. Since viscosity is generally correlated with the presence of interactions (van der Waals and hydrogen bonding), we can explain the higher value for DES_LA1 by the amount of hydrogen bonding network between choline chloride and lactic acid in a molar ratio 1:1, which ensures better compaction at this ratio, hence lower mobility.

The viscosity data for DES_GA3 found in the literature [55] are given in the form of the linear dependence of lnη on 1/T, which leads to lower values than ours, because they probably contain a higher percentage of water (of 5.25%) than ours (of 2%).

FTIR spectra of pure DES_GA3 indicates the formation of NaDES structure between components from the shift in the peak representing the C=O vibration in GA at 1728/1730 cm^−1^ but also from the lack of the choline chloride band at 1349 cm^−1^ (Figure S5).

The detailed interpretation of the FTIR spectra for NaDES can be found in the literature and confirms the presence of eutectic mixtures between choline chloride and lactic/glycolic acids [22,30,56].

## 4. Conclusions

In this study, chitosan-based active films plasticized with a natural deep eutectic solvent (NaDES) composed of choline chloride and glycolic acid (1:3), and incorporating hawthorn extract, were successfully developed using the direct casting method. Increasing the NaDES content from 44 to 70 wt% led to pronounced reduction in Young’s modulus (from ~176 to 12 MPa), while tensile strength decreased by 44%, improving film flexibility and confirming the effective plasticizing role of DES_GA3 within the chitosan matrix. Higher NaDES concentrations also reduced the films’ thermal stability. The formulation containing 50 wt% NaDES exhibited the best balance between mechanical strength and barrier performance A significant reduction in Young’s modulus (*p* < 0.05) was observed for 50DES_GA3_EH film, showing a 77% decrease relative to extract-free counterparts. The relatively small WVP values obtained for DES_GA3 films (~4 × 10^−11^ g·m^−1^s^−1^Pa^−1^) offer a favourable premise for the development of chitosan films intended for dry-food packaging. These effects arise from hydrogen-bonding interactions between chitosan chains and NaDES components, which, together with the smaller glycolic acid molecules, probably promote the formation of a denser and more cohesive CS–NaDES network, thereby lowering the water vapour permeability of the resulting films. Moreover, the chitosan–NaDES films exhibited pronounced UV absorption, which further extended into the UVA–visible region upon extract incorporation, consistent with their twofold increase in DPPH free radical scavenging activity. Overall, these findings demonstrate that chitosan–NaDES systems, particularly those incorporating natural extracts, represent promising, sustainable, and eco-friendly materials with great potential for active food coating and biodegradable packaging applications.

## Figures and Tables

**Table 1:** Variation in MC and WS with NaDES content for CS films. Different letters indicate statistically significant differences between films (*p* < 0.05).

Sample	MC, %	WS, %
44DES_GA3	17.5 ± 0.2 ^d^	31.9 ± 1.3 ^e^
50DES_GA3	19.5 ± 0.3 ^c^	34.8 ± 0.4 ^d^
60DES_GA3	21.2 ± 0.7 ^b^	43.3 ± 0.5 ^b^
70DES_GA3	24.7 ± 1.3 ^a^	50.5 ± 1.0 ^a^
50DES_GA3_EH	20.0 ± 1.2 ^bc^	35.7 ± 1.3 ^c^

**Table 2:** The antibacterial activity of the analyzed films and extracts on targeted bacteria.

Acronym	IZ (Inhibition Zone), mm	
	*E. coli* (−)	*B. subtilis* (+)
50DES_GA3	nd	0.07 ± 0.13 ^d^
Ex_EH_GA3	4.33 ± 1.52 ^a^	4.33 ± 0.57 ^a^
50DES_GA3_EH	1.66 ± 0.33 ^c^	nd
50DES_LA1	nd	2.33 ± 0.57 ^b^
Ex_EH_LA1	3.66 ± 0.57 ^b^	1.5 ± 0.5 ^c^
50DES_LA1_EH	nd	nd

nd—not detected; different lowercase letters indicate statistically significant differences between films and extracts (*p* < 0.05).

**Table 3:** Principal values of thermal analysis for DES_GA3.

Endo I (°C)	Mass loss (%) RT-220 °C	Exo I (°C)	Endo II (°C)	Mass loss (%) 220–270 °C	Mass loss (%) 300–600 °C	Exo II (°C)
70.8	16.0	227.4	268.0	78.3	5.6	488.8

## Data Availability

The original contributions presented in this study are included in the article/Supplementary Materials. Further inquiries can be directed to the corresponding author.

## Supplementary Materials

The following supporting information can be downloaded at: https://www.mdpi.com/article/10.3390/polym17243250/s1, Figure S1: Flowchart for NaDES preparation, extract addition and CS-films formation; Figure S2: FTIR maps for chitosan-NaDES films; red areas indicate higher absorbance, while blue areas—lower absorbance; Figure S3: Appearance of CS-NaDES films; Figure S4: Stress–strain curves of CS-NaDES films (red-44DES_GA3; blue-50DES_GA3; green-60DES_GA3; grey-70DES_GA3, purple-50DES_GA3_EH); Figure S5: FTIR spectra of the studied DES_GA3 and its constituent compounds, glycolic acid (GA) and choline chloride (ChCl); Table S1: The quantities of components used to prepare CS–NaDES films; Table S2: Principal values of thermal analysis for chitosan-DES_GA3 films.
