# Supplementary material for: Formulation and Characterization of Chitosan Films Incorporating Hawthorn Polyphenolic Extracts via Natural Deep Eutectic Solvents
Source: Polymers (Basel). 2025 Dec 6;17(24):3250. doi: 10.3390/polym17243250 (PMC12737212; doi:10.3390/polym17243250)
Supplement: Supplementary file 1 [file polymers-17-03250-s001.zip › polymers-4006199-supplementary.pdf]

Formulation and Characterization of Chitosan Films Incorporating Hawthorn Polyphenolic Extracts via Natural Deep Eutectic Solvents

Oana Ciocirlan[1]*, Adina Gavrila[1], Gabriela Isopencu[1], Ludmila Motelica[2,3], Ovidiu-Cristian Oprea[1,3], Adrian Ionut Nicoara[1,4], Sergiu Sima[1], Paul Stanescu[1]

[1]*Faculty of Chemical Engineering and Biotechnologies, National University of Science and Technology Politehnica Bucharest, 132 Calea Grivitei, 010737, Bucharest, Romania;* adina.gavrila@upb.ro (A.G.); gabriela.isopencu@upb.ro (G.I.); ovidiu.oprea@upb.ro (O.O.); adrian.nicoara@upb.ro (A.I.N.); sergiu.sima@upb.ro (S.S.); paul.stanescu@upb.ro (P.S.)
[2]*Advanced Research Center for Innovative Materials, Products and Processes, National University of Science and Technology POLITEHNICA Bucharest, 313 Splaiul Independentei, 060042, Bucharest, Romania;* ludmila.motelica@upb.ro
[3]*Academy of Romanian Scientists, 3 Ilfov Street, 050044 Bucharest, Romania.*
[4]*Department of Science and Engineering of Oxide Materials and Nanomaterials, National Research Center for Micro and Nanomaterials, 313 Spl. Independenţei, 060042 Bucharest, ROMANIA*

**Correspondence: oana.ciocirlan@upb.ro*

Table S1. The quantities of components used to prepare CS–NaDES films

Film acronim	m_{DES}, g	m_{CS}, g	m_{DES}/m_{CS}	m_{water}, g	% solids (w/v)	% CS (w/v)	NaDES–extractant solutions, g	% DES in film (w/w)
44DES_GA3	0.42	0.53	0.79	49	1.9	1.0	0	44
50DES_GA3	0.5	0.5	1.00	49	2.0	1.0	0	50
60DES_GA3	0.6	0.4	1.50	49	2.0	0.8	0	60
70DES_GA3	0.7	0.3	2.33	49	2.0	0.6	0	70
50DES_GA3_ EH	0	0.5	1.00	49	2.0	1.0	1.67	50
50DES_LA1	0.5	0.5	1.00	49	2.0	1.0	0	50
50DES_LA1_ EH	0	0.5	1.00	49	2.0	1.0	1.67	50

Table S2. Principal values of thermal analysis for chitosan-DES_GA3 films

Sample	Mass loss (% RT-170°C	Endo I (°C)	Endo II (°C)	Mass loss (%) 170-400°C	Mass loss (%) 400-600°C	Exo (°C)
44DES_GA3	10.5	87.9	263.0	55.2	34.3	619.8
50DES_GA3	11.0	70.3	259.0	54.4	34.6	590.0/612.9
60DES_GA3	9.2	110.3	265.1	63.6	27.3	581.6
70DES_GA3	9.8	78.4	253.0	60.7	27.3	577.7
50DES_GA3_EH	11.1	70.2	264.0	54.0	35.8	596.1

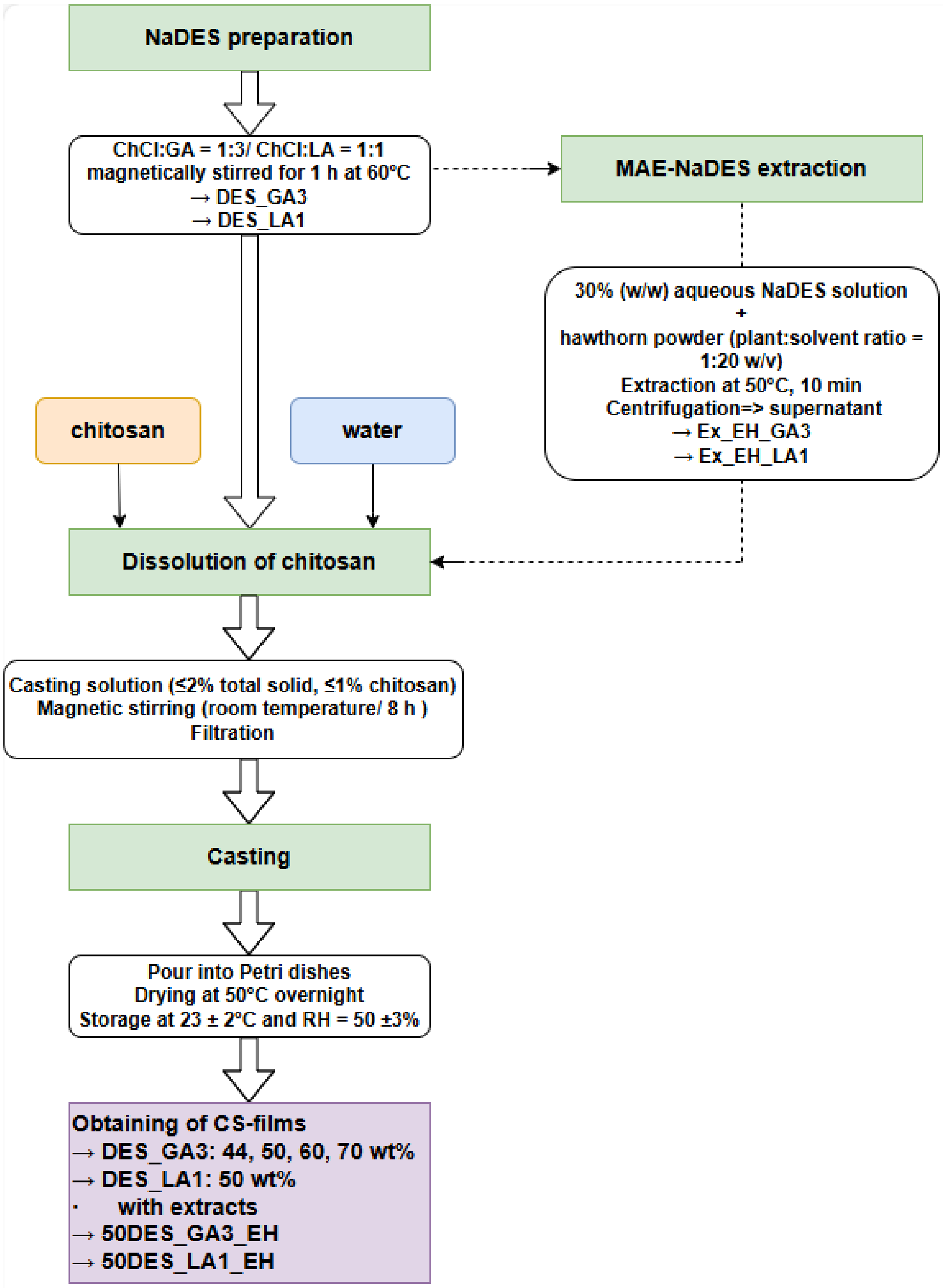

Figure S1. Flowchart for NaDES preparation, extract addition and CS-films formation

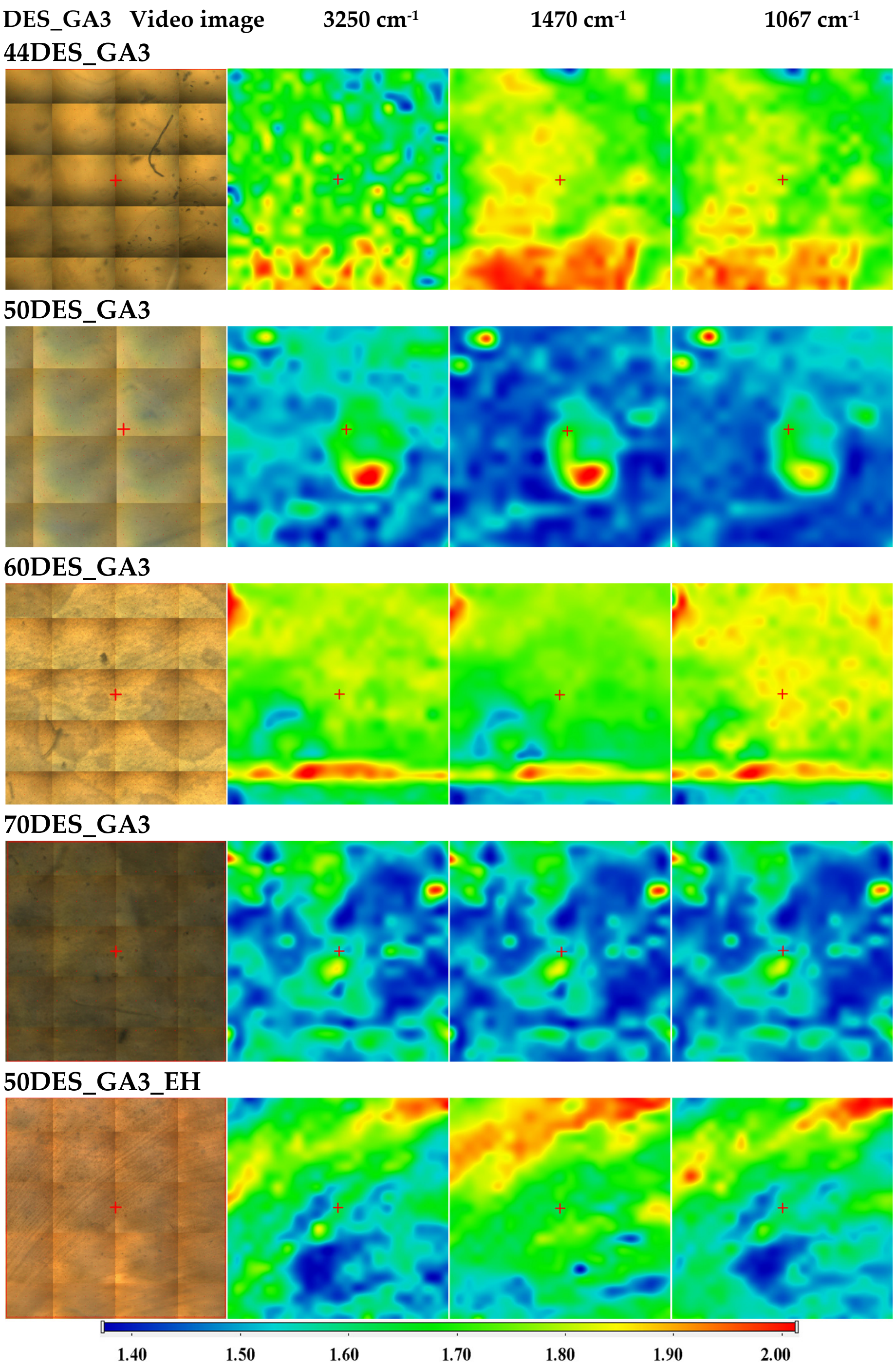

Figure S2. FTIR maps for chitosan-NaDES films; red areas indicate higher absorbance, while blue areas, lower absorbance

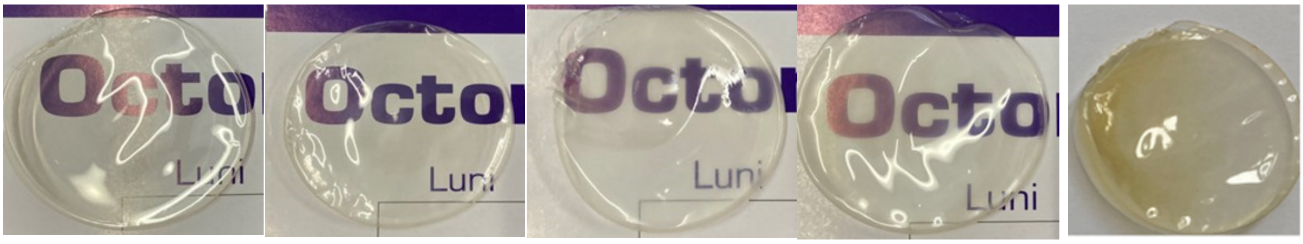

44DES_GA3 50DES_GA3 60DES_GA3 70DES_GA3 50DES_GA3_EH

Figure S3. Appearance of CS-NaDES films

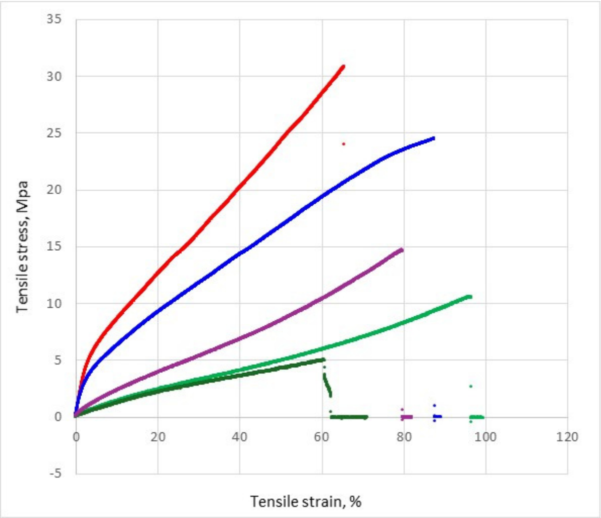

Figure S4. Stress-strain curves of CS-NaDES films (red-44DES_GA3; blue-50DES_GA3; green-60DES_GA3; grey-70DES_GA3, purple-50DES_GA3_EH)

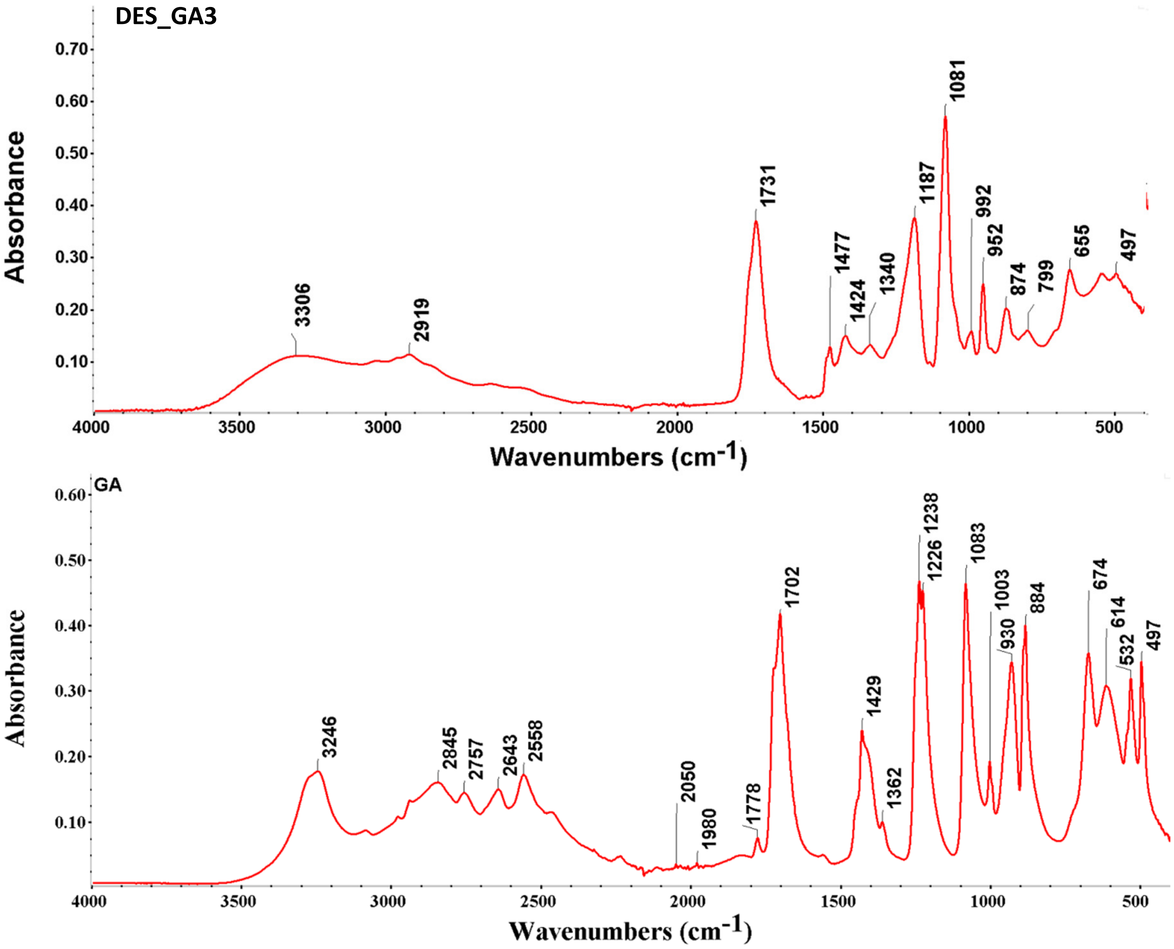

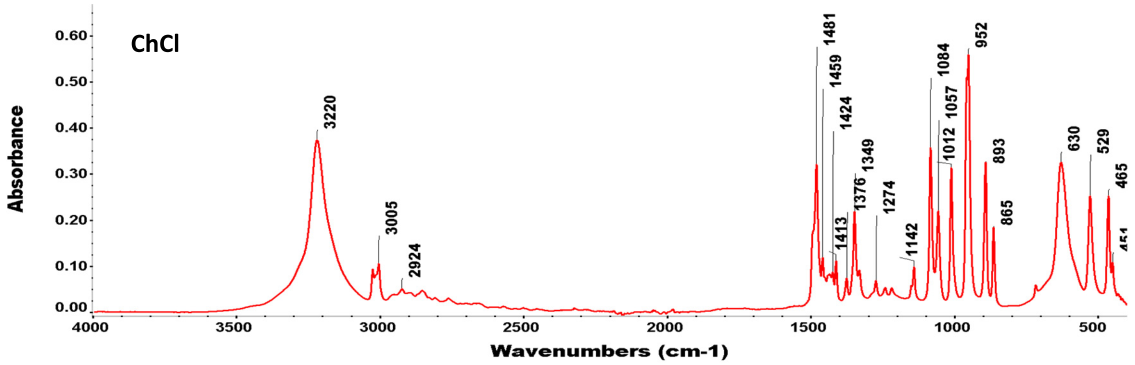

Figure S5. FTIR spectra of the studied DES_GA3 and its constituent compounds, glycolic acid (GA) and choline chloride (ChCl)
